# Family Caregiver Needs and Preferences for Virtual Training to Manage Behavioral Symptoms of Dementia

**DOI:** 10.1093/geroni/igab046.007

**Published:** 2021-12-17

**Authors:** Maggie Ramirez, Miriana Duran, Chester Pabiniak, Kelly Hansen, James Ralston, Susan McCurry, Linda Teri, Robert Penfold

**Affiliations:** 1 University of Washington School of Public Health, Seattle, Washington, United States; 2 Kaiser Permanente Washington Health Research Institute, Seattle, Washington, United States; 3 University of Washington School of Nursing, Seattle, Washington, United States; 4 University of Washington, Seattle, Washington, United States

## Abstract

STAR-Caregivers Virtual Training and Follow-up (STAR-VTF) is adapted from an evidence-based, in-person program that teaches family caregivers to manage behavioral and psychological symptoms of dementia (BPSD). The study objective was to understand the needs of family caregivers for improving BPSD management and the extent to which caregivers perceived that STAR-VTF could address those needs. We conducted 15 semi-structured interviews with family caregivers of people with dementia. We showed caregivers prototypes of STAR-VTF online self-directed materials. We obtained caregiver feedback, focusing on needs and preferences and perceived barriers to using STAR-VTF. We used a hybrid approach of inductive and deductive coding and aggregated codes to develop themes. The idea of a virtual training program for learning to manage BPSD appealed to caregivers. They said healthcare providers did not provide adequate education in the early disease stages about the personality and behavior symptoms that can affect people with dementia. Caregivers found it unexpected and frustrating when the person with dementia began experiencing BPSD, symptoms they felt unprepared to manage. Accordingly, caregivers expressed a strong desire for the healthcare organization to offer programs such as STAR-VTF much sooner. Many were interested in the virtual aspect of the training due to the convenience of receiving help from home and the perception that help from a virtual program would be timelier than traditional service modalities. Given caregivers’ limited time, they suggested dividing the STAR-VTF content into chunks to review as time permitted. Caregivers reported a preference for having the same coach for the program duration.

